# Genome-Wide Identification and Characterization of bHLH Family in *Kandelia obovata* Under Salt Stress

**DOI:** 10.3390/life16081247

**Published:** 2026-07-28

**Authors:** Zhixia Zhao, Huizi Liu, Sheng Yang, Xing Liu, Qiuxia Chen, Jinwang Wang

**Affiliations:** 1 Zhejiang Institute of Subtropical Crops, Zhejiang Academy of Agricultural Sciences, Wenzhou 325005, China; zhaozx818@126.com (Z.Z.); 13125982715@163.com (H.L.); yangs@zaas.ac.cn (S.Y.); liuxingzy1989@163.com (X.L.); yzscqx@163.com (Q.C.); 2 Engineering Research Center for Southeast Coastal Characteristic Plants of National Forestry and Grassland Administration, Wenzhou 325005, China; 3 Wenzhou Key Laboratory of Resource Plant Innovation and Utilization, Wenzhou 325005, China

**Keywords:** *Kandelia obovata*, basic helix–loop–helix, expression pattern, salt stress, co-expression network

## Abstract

**Background/Objectives**: bHLH transcription factors regulate key plant processes including development, stress adaptation, and metabolism. In *Kandelia obovata*, a mangrove species well known for its high tolerance to harsh environments, the bHLH gene family has not been systematically investigated. Here, this study aims to perform genome-wide identification and expression profiling of *KobHLH* genes under normal and saline conditions. **Methods**: bHLH members were identified by homology searches (HMMER and BLASTp) against the *K. obovata* protein dataset and verified with SMART-based domain analysis. Subsequent analyses covered physicochemical features, conserved motif composition, and phylogenetic relationships. Transcript abundance was quantified via RNA-seq in various organs (roots, stems, leaves, flowers, and fruits) as well as under saline conditions. Differentially expressed transcripts were selected for co-expression network construction. **Results**: Phylogenetic analysis of the 118 identified *KobHLH* loci assigned them into 18 distinct clades. Organ-preferential expression patterns were observed, leading to their classification into three putative functional categories. Upon salinity exposure (10–30‰), 61 of the 118 KobHLH genes exhibited differential expression, grouped into ten significant clusters. A co-expression network comprising 20 hub genes and 198 edges was constructed. **Conclusions**: This study provides the first genome-wide characterization of the *bHLH* family in *K. obovata*, revealing its evolutionary divergence and identifying 61 salt-responsive candidates, notably KobHLH108 and KobHLH5 associated with secondary metabolism, for further functional validation. The findings provide a foundation for future functional studies on stress adaptation mechanisms in this ecologically valuable mangrove species.

## 1. Introduction

Transcription factors (TFs) regulate various processes in plant growth and development [1]. Despite their limited numbers, TFs can generate diverse cell types through combinatorial regulation [2,3,4,5,6,7]. The basic helix–loop–helix (bHLH) proteins are one of the largest TF families and are widespread among eukaryotes [8,9]. Since the first bHLH TF was discovered in animals [10], numerous bHLH genes have been identified, revealing conserved structural features. The bHLH TFs are defined by their characteristic bHLH domain of approximately 60 amino acids, which comprises a basic N-terminal region responsible for DNA binding as well as a C-terminal HLH region that mediates protein–protein interactions [11]. The basic region uses the conserved residues (Glu and Arg-16) to recognize the canonical enhancer-box (E-box, 5′-CANNTG-3′), while His/Lys-9 and Arg-7 recognize non-canonical E-box sequences, such as the G-box (5′-CACGTG-3′) [12]. In addition, nucleotides flanking the core motif also modulate binding affinity [13,14,15,16]. Meanwhile, the HLH region contains two amphiphilic lipid alpha helices, composed mainly of hydrophobic residues and connected by loops of variable length and sequence [17]. Furthermore, cocrystal structural analysis suggests an additional conserved motif in bHLH proteins [13,18].

The bHLH TF account for a substantial proportion of the plant genome and are extensively involved in stress responses and developmental regulation [19,20]. Specifically, their functions span a broad range of developmental processes including seed germination [21], pistil [22,23], pollen [24,25], trichome [26,27], embryo [28], shoot branching [29], leaf [30,31], stomatal [32,33], root [34,35], fruit, and flower development [36,37,38,39,40]. bHLHs also participate in light [41,42,43] and phytohormone [44,45,46] signaling. For example, PIF3 is required for photo-induced signal transduction [41] and PRE1 overexpression activates the gibberellin-dependent pathway [45]. bHLH TFs are also implicated in plant responses to abiotic stressors, including drought [44,47], cold [48,49], heat [50], and salt stress [51,52]. Notably, *OsbHLH068* (*Oryza sativa*) and *AtbHLH112* (*Arabidopsis thaliana*) are active in the salt-stress response [53], *AtbHLH112* acts as a transcriptional activator in drought [54], *AtbHLH116* (*ICE1*) enhances cold tolerance [47], and a set of gene (*AtbHLH17*, *AtNAC002*, *AtNAC072*, *OsbHLH006*, *OsbHLH148*, *TabHLH39*, *PtrbHLH186*, *PtrNAC006*, *PtrNAC007*, and *PtrNAC120*) confer drought tolerance [51,55,56,57,58,59,60,61]. Furthermore, bHLH TFs play critical roles in regulating the accumulation of secondary metabolites and influencing biosynthesis [17,62].

Mangroves hold ecological and commercial value, are distributed across 118 countries and territories, and collectively cover approximately1.7 million hectares [63]. *Kandelia obovata* (*Rhizophoraceae*) is one of the most stress-resistant mangrove species and is extensively employed in coastal wetland ecological restoration, owing to its viviparous reproduction, ornamental appearance, and unique floral morphology [64]. As halophytes native to coastal intertidal zones, mangroves are routinely subjected to ultraviolet radiation, hypoxia, and high salinity [65]. According to the Intergovernmental Panel on Climate Change (IPCC) projections, 30–40% of coastal wetlands and up to 100% of mangroves could be lost over the next 100 years if the current rate of loss continues. Salinity is the predominant abiotic factor limiting mangrove growth and development. Although mangroves are ecologically vital, the bHLH transcription factor family, which serves as a key regulator of stress responses in plants, has not been systematically investigated in any mangrove species, including *K. obovata*. Therefore, a comprehensive understanding of the molecular mechanism underlying abiotic stress tolerance in mangroves is essential for the effective conservation of these ecologically significant resources.

In this study, we performed a genome-wide survey of the *bHLH* gene family in *K. obovata*, identifying 118 members and analyzing their chromosomal locations, conserved motifs, evolutionary relationships, functions, and expression patterns. Furthermore, we constructed a co-expression network for *KobHLHs* and other genes under salt stress, comprising 20 gene nodes and 198 correlation edges. The results enhance the current understanding of bHLH TFs in mangrove salt tolerance and offer a valuable genetic resource for further research on *K. obovata*.

## 2. Materials and Methods

### 2.1. Sequence Retrieval from Plant Genomes

The CDS, protein coding sequence, whole-genome sequence, and annotation information for 24 plants species, including A. thaliana, *B. distachyon*, *B. oleracea*, *B. rapa*, *C. quinoa*, *G. max*, *C. reinhardtii*, *C. sativus*, *G. raimondii*, *H. vulgare*, *O. sativa*, *O. lucimarinus*, *P. vulgaris*, *P. patens*, *P. trichocarpa*, *S. moellendorffii*, *S. italica*, *S. lycopersicum*, *S. tuberosum*, *S. bicolor*, *T. cacao*, *V. vinifera*, *V. carteri*, and *Z. mays*, were downloaded from the Joint Genome Institute (JGI; https://phytozome-next.jgi.doe.gov, accessed on 12 October 2023). The CDS and protein-coding sequence of *K. obovata* were downloaded from the CNCB Genome Warehouse (https://download.cncb.ac.cn/gwh/Plants/Kandelia_obovata-Kandelia_candel_GWHACBH00000000.1/, accessed on 12 October 2023).

### 2.2. Identification and Characterization of bHLH Genes

The bHLH HMM profile (PF00010) was downloaded from the Pfam database (http://pfam.xfam.org, accessed on 15 October 2023). The *A. thaliana* bHLH sequence was obtained from The Arabidopsis Information Resource (TAIR) (www.arabidopsis.org, accessed on 16 October 2023). Notably, 158 of the 161 annotated A. thaliana bHLH genes were downloaded from https://www.arabidopsis.org/browse/genefamily/bHLH.jsp (accessed on 18 October 2023), whereas *At4g38071*, *At4g49770*, and *At2g20095* were absent from the *A. thaliana* genome (tair10/TAIR10_cdna_20101214).

To identify bHLH candidates in *K. obovata*, a two-step computational approach was employed, followed by domain verification. First, HMMER v3.3 [65] was used with default parameters to search the *K. obovata* protein dataset against the Pfam bHLH profile (PF00010), using an E-value threshold of 1 × 10^−5^. Second, a BLASTp [66] was performed against the *K. obovata* protein data with the *A. thaliana* bHLH sequences, with an E-value threshold of 1 × 10^−5^ and default settings for other parameters.

The candidate set was defined as the union of HMMER and BLASTp hits. Each candidate was then subjected to domain verification using SMART (http://smart.embl-heidelberg.de/, accessed on 18 October 2023) [67] with the default E-value threshold. Sequences lacking a detectable HLH domain were removed. Ultimately, 118 non-redundant bHLH genes were identified in the *K. obovata* genome.

**Figure 1:**
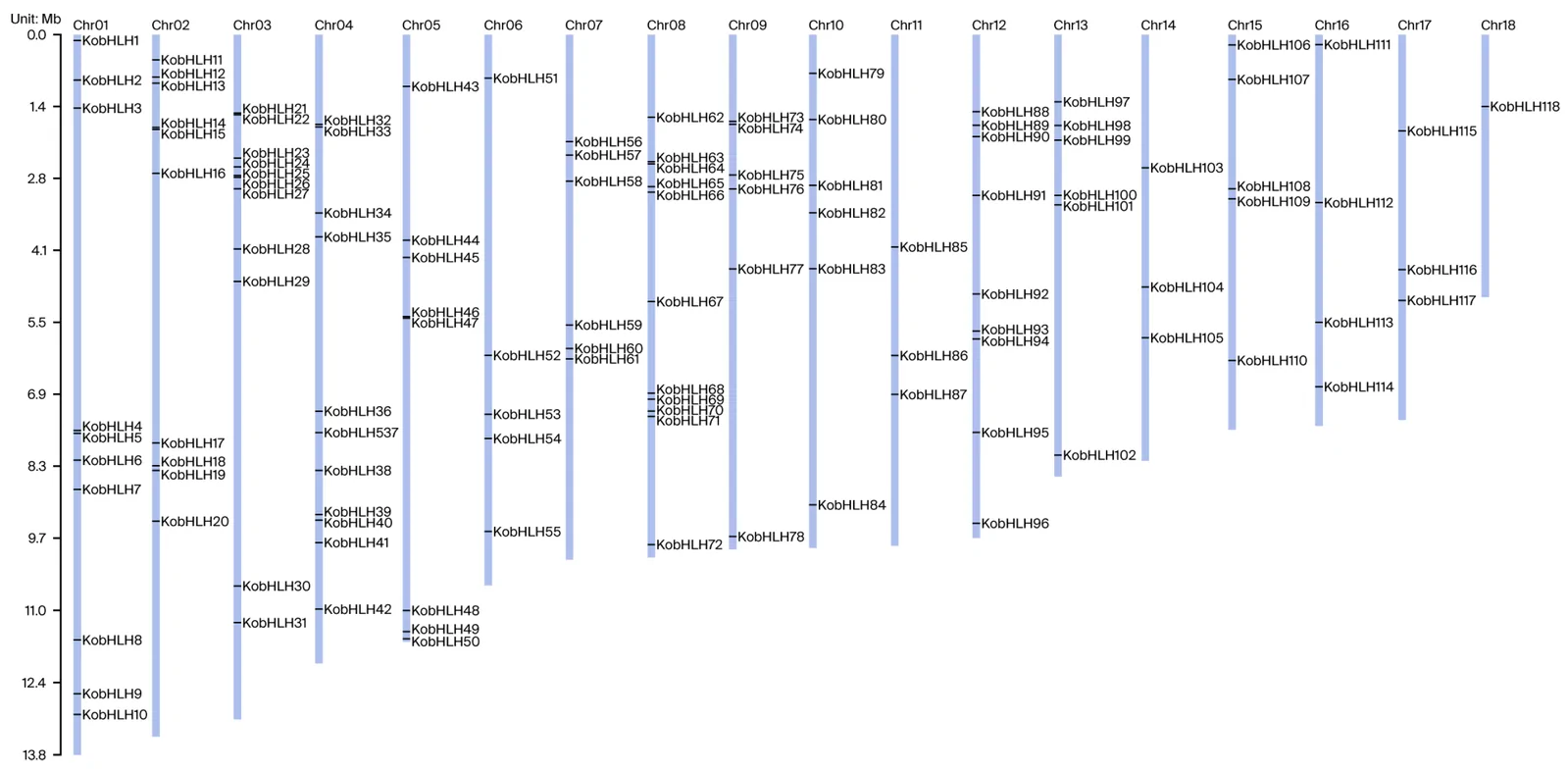
Chromosomal distribution of *bHLH* genes in *K. obovata*. A total of 118 *bHLH* genes were mapped to 18 chromosomes, with each vertical line representing a chromosome and its length scaled to chromosome size.

These genes were systematically named *KobHLH1*–*KobHLH118* according to their physical order along chromosomes 1–18 (Figure 1, Table S1).

### 2.3. Physicochemical Properties, Chromosomal Location, and Evolutionary Analysis of the bHLH Gene Family

The physicochemical properties, including length, molecular weight(MW), and isoelectric point(PI) of the KobHLH proteins, were determined using ProtParam [4,68]. Subcellular localization was predicted with ProtComp ver. 9.0 (http://linux1.softberry.com/berry.phtml?topic=protcomppl&group=programs&subgr9.0oup=proloc, accessed on 20 October 2023). DNA-binding domains of the *KobHLH* superfamily members were analyzed using the SMART database. Chromosomal locations of *KobHLH* genes were visualized with TBtools-II v2.030 [69]. To infer evolutionary relationships, a phylogenetic tree was generated for 25 plant species with Ortho Finder v2.2.7 [70] and plotted using ggtree v1.14.16. The numbers of *bHLH* genes in each species were obtained from previous studies [71,72,73,74,75,76,77,78,79,80,81] and displayed in a bar chart.

### 2.4. Phylogenetic and Alignment Analyses

To infer phylogenetic relationships, bHLH protein sequences of *A. thaliana* and *K. obovata* were aligned using MAFFT v7.450 [82,83,84]. A maximum-likelihood phylogenetic tree was then constructed using IQ-TREE v2 [85] with the optimal substitution model determined by ModelFinder and branch support estimated by ultrafast bootstrapping (1000 replicates). Then, the phylogenetic tree was visualized using ggtree. Multiple sequence alignment of the HLH amino acid sequences was performed using ClustalW 1.7 with default parameters [86,87], and the aligned results were visualized with BioEdit ver. 7.2.5 [88].

### 2.5. Motif and Conserved Domain Analysis

The bHLH protein sequences from *K. obovata* were aligned using MAFFT, and the maximum-likelihood (ML) phylogenetic tree was constructed using FastTree v2.2.0. Conserved motifs in KobHLH proteins were detected using MEME v5.4.1 (http://memesuite.org/, accessed on 21 October 2023) [89], and conserved domains of the KobHLH proteins were predicted via Batch CD-search [90] (https://www.ncbi.nlm.nih.gov/Stru-cture/bwrpsb/bwrpsb.cgi, accessed on 21 October 2023). Then the conserved domain and ML tree were visualized using TBtools. The exon–intron structures of the *KobHLH* genes were analyzed with the Gene Structure Display Server (GSDS; http://GSDS.cbi.pku.edu.cn, accessed on 21 October 2023) [91].

### 2.6. Cis-Elements and Collinearity Analysis

The 2 Kb upstream promoter regions of each *KobHLH* gene were submitted to PlantCARE (http://bioinformatics.psb.ugent.be/webtools/plantcare/html/, accessed on 22 October 2023) for cis-acting elements prediction [92]. Collinearity relationships among *K. obovata*, *A. thaliana*, and *P. trichocarpa* were examined using MCScanX v1.1 with default parameters [93]. *KobHLH*, *AtbHLH*, and *PtrbHLH* gene pairs were colored red, blue, and green, respectively, while inter-species pairs were shown in gray. Tandem duplication events were defined as homologous KobHLH copies located in close proximity on the same chromosome with no more than five intervening genes. Segmental duplication events were identified as KobHLH paralogs located within collinear blocks detected by MCScanX v1.1. The number of genes derived from each duplication type was tallied, and their contribution to expansion of the *KobHLH* family was assessed.

### 2.7. Expression Profiling of KobHLH Genes in Tissues and Under Salt Stress

To examine tissue-specific expression, transcriptome data for organs, including root, stem, leaf, flower, pistil, stamen, sepal, and fruit, were retrieved from NCBI under accession number PRJNA678025.

Transcriptome data for salt-stress response analysis were downloaded from NCBI (PRJNA1051781), derived from root tissues of *K. obovata* subjected to salinity treatments. Briefly, mature hypocotyls of *K. obovata* were collected from a germplasm garden along the Aojiang River, China, and planted at a depth of 10 cm in simulation troughs filled with 30 cm of river silt. Plants were cultivated at 21 °C–25 °C and treated with 0‰ (control), 10‰, 20‰, and 30‰ NaCl up to 6 months, with three independent biological replicates per treatment. Roots were harvested for RNA-seq. For heatmap visualization, log10-transformed FPKM values were z-score normalized across samples, then clustered using Pearson distance with complete linkage (pheatmap v1.0.12 R package). Differential expression between each salt treatment (S1, S2, S3) and the control (S0) was analyzed using the Majorbio Cloud Platform 2024, with genes meeting |log_2_(fold change)| ≥ 1 and FDR-adjusted *p*-value < 0.05 designated as differentially expressed. Hierarchical clustering of *KobHLH* genes was performed using Pearson distance and complete linkage. Group assignments for tissue-specific (Section 3.8) and salt-responsive (Section 3.9) expression patterns were determined by visual inspection of the dendrogram, and clustering robustness was verified by cross-comparison across independent salt concentrations.

### 2.8. Co-Expression Network Analysis

Salinity-responsive DEGs were identified from pairwise comparisons (S1_vs_S0, S2_vs_S0, S3_vs_S0) using the transcriptome dataset (PRJNA1051781). DEGs were defined as those with |log_2_FC| ≥ 1 and *p*-value < 0.05. FPKM values were used to calculate the correlation and *p*-value between each *KobHLH* gene and all other DEGs. Pairs with absolute correlation values > 0.98 and *p* < 0.001 were selected as co-expressed and were visualized with Cytoscape v3.6.0 [94]. A conservative correlation threshold (|r| > 0.98) was adopted to reduce the likelihood of false-positive associations arising from multiple comparisons within the DEG set, thereby prioritizing the most confident interactions for downstream interpretation. To assess the biological relevance of the network, hub genes identified in the co-expression network were compared against the KEGG pathway enrichment results (Section 2.9).

### 2.9. KEGG Pathway Enrichment Analysis

The Kyoto Encyclopedia of Genes and Genomes (KEGG) pathway enrichment analysis was performed using the Majorbio Cloud Platform (www.majorbio.com, accessed on 22 October 2023). Pathways with *p*-value < 0.05 were considered significantly enriched. Subsequently, enrichment results were compared across experimental groups to determine the genes and pathways most relevant to the salt-stress response of *K. obovata*.

## 3. Results

### 3.1. Genome-Wide Identification of the bHLH Gene Family in K. obovata

We identified 118 bHLH genes in the *K. obovata* genome and named them *KobHLH1-KobHLH118* sequentially according to their physical positions from the top of chromosome 1 to the bottom of chromosome 18 (Figure 1, Table S1). Table S1 presents the chromosome numbers, protein lengths, isoelectric points (PIs), molecular weights (MWs), and predicted subcellular locations (W) for each KobHLH protein. The greatest number of genes were found on chromosomes 3, 4, and 8, while only one gene was located on chromosome 18 (Figure 1, Table S1). *KobHLH42* exhibited the shortest protein length (90 amino acids) and the lowest MW (10.3475 kDa), whereas KobHLH115 exhibited the longest protein length (962 amino acids) and the highest MW (113.39 kDa) (Table S1). The PIs ranged from 4.72 (KobHLH53) to 11.43 (KobHLH34), and 90% of KobHLH proteins were predicted to be nucleus-localized (Table S1).

### 3.2. Phylogenetic Relationships of bHLH Genes Across Plant Species

**Figure 2:**
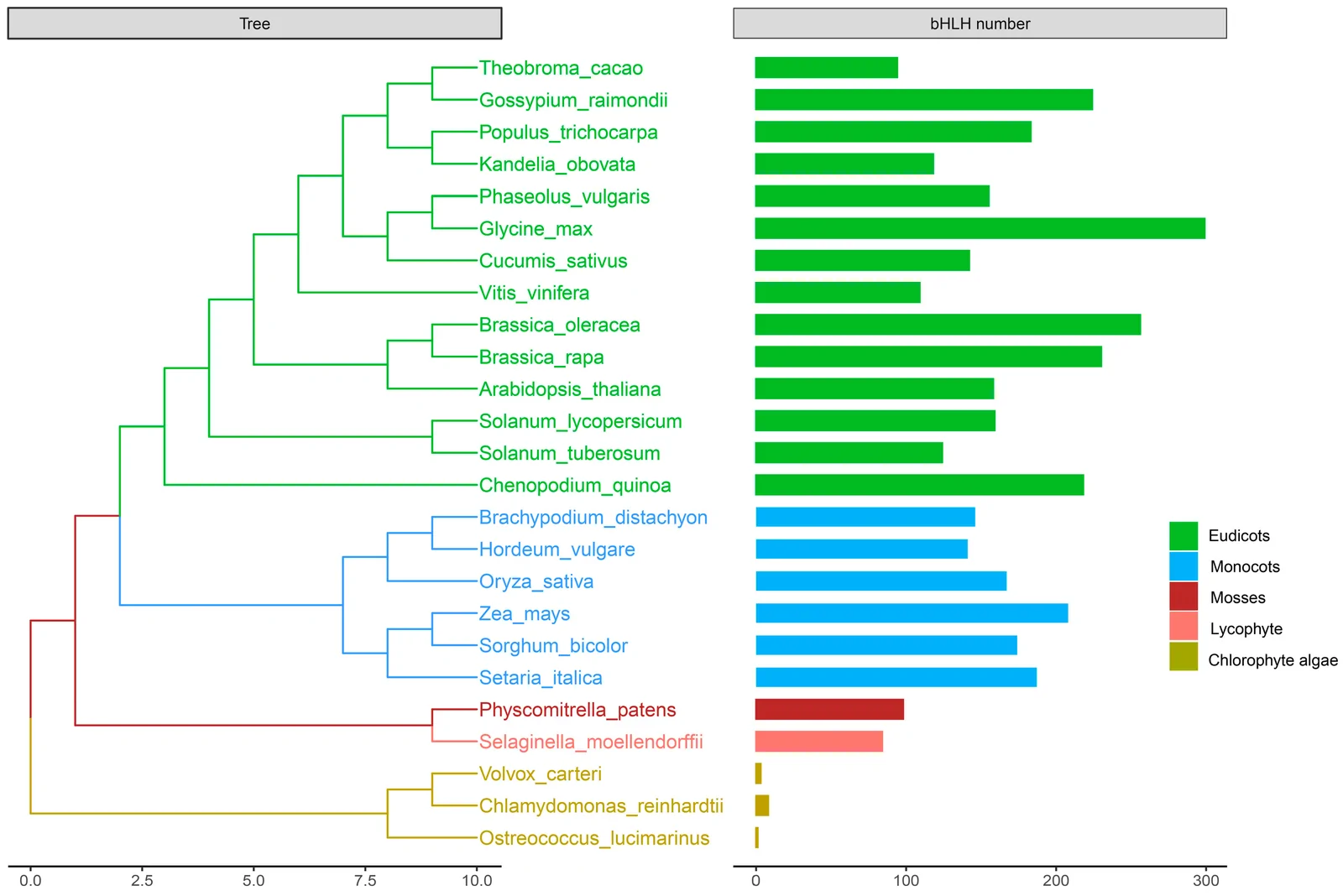
Phylogenetic relationships of the *bHLH* gene family across 25 plant species. Shown are *bHLH* genes counts for 25 plant species, including 14 eudicots (*Kandelia obovata*, *Theobroma cacao*, *Gossypium raimondii*, *Populus trichocarpa*, *Phaseolus vulgaris*, *Glycine max*, *Cucumis sativus*, *Vitis vinifera*, *Brassica oleracea*, *Brassica rapa*, *Arabidopsis thaliana*, *Solanum lycopersicum*, *Solanum tuberosum*, and *Chenopodium quinoa*), 6 monocots (*Brachypodium distachyon*, *Hordeum vulgare*, *Oryza sativa*, *Zea mays*, *Sorghum bicolor*, and *Setaria italica*), 1 lycophytes (*Selaginella moellendorffii*), 3 chlorophyte algae (*Volvox carteri*, *Chlamydomonas reinhardtii*, and *Ostreococcus lucimarinus*), and 1 mosses (*Physcomitrella patens*).

In order to explore the evolutionary relationships between the bHLH family in *K. obovata* and those of other plant species, we constructed a phylogenetic tree encompassing 25 species, including 14 dicots (*K. obovata*, *Theobroma cacao*, *Gossypium raimondii*, *Populus trichocarpa*, *Phaseolus vulgaris*, *Glycine max*, *Cucumis sativus*, *Vitis vinifera*, *Brassica oleracea*, *Brassica rapa*, *A. thaliana*, *Solanum lycopersicum*, *Solanum tuberosum*, and *Chenopodium quinoa*), six monocots (*Brachypodium distachyon*, *Hordeum vulgare*, *O. sativa*, *Zea mays*, *Sorghum bicolor*, and *Setaria italica*), one moss (*Physcomitrella patens*), one lycophyte (the *lycopodium Selaginella moellendorffii*) and three chlorophyte algae (*Volvox carteri*, *Chlamydomonas reinhardtii*, and *Ostreococcus lucimarinus*) (Figure 2). The 25 species were well-distributed across five major taxonomic clades, with the dicots and monocots clustering more closely to each other than to the chlorophyte algae, as expected. Among the 14 dicots, bHLH family member counts ranged from 94 to 299, and *K. obovata* was found to harbor 118 bHLH family members. Notably, *K. obovata* was grouped within the same clade as *P. trichocarpa*, a representative woody plant, suggesting that they may share a common evolutionary origin.

### 3.3. Phylogenetic Analysis of bHLH Proteins in K. obovata and A. thaliana

**Figure 3:**
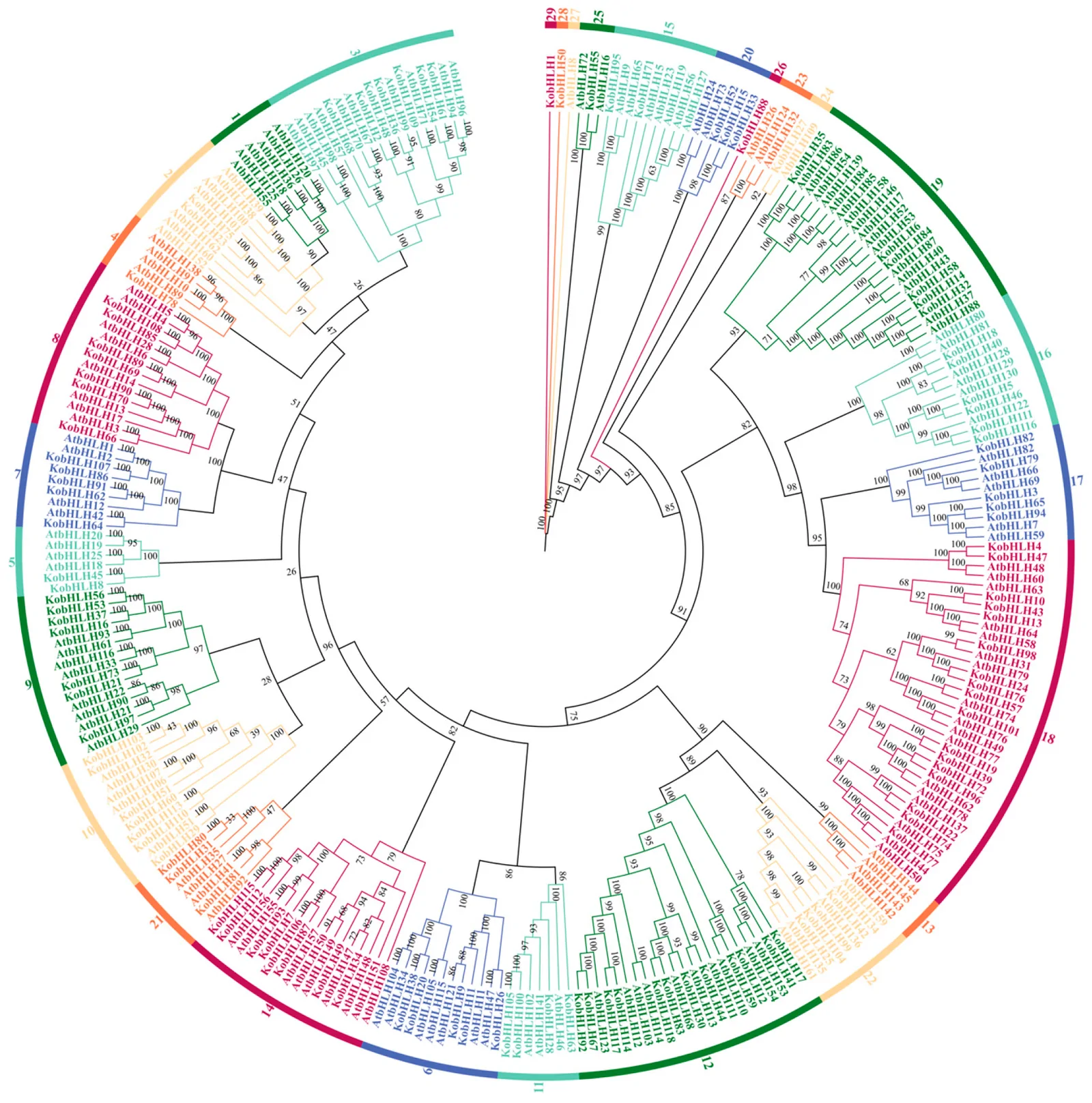
Phylogenetic tree of bHLH proteins from *K. obovata* and *A. thaliana*. Subfamilies (numbered 1–23) are coded according to the *A. thaliana* classification system [17,95]. Node values indicate ultrafast bootstrap support percentages (1000 replicates).

In order to explore the evolutionary relationships between bHLH proteins in K. obovata and A. thaliana, we constructed a maximum-likelihood (ML) phylogenetic tree based on bHLH amino acid sequences of 118 KobHLH and 158 AtbHLH proteins (Figure 3), using the classification framework established for *A. thaliana* [17,95]. The tree resolved the proteins into 23 subfamilies. AtbHLH members were distributed across all 23 subfamilies, whereas KobHLH members were found in only 18, with no representatives in subfamilies 2, 13, 20, 21, and 23. Subfamily assignment for each KobHLH protein was determined by its phylogenetic grouping with the nearest AtbHLH ortholog, and was further corroborated by conserved motif composition and exon–intron structure. The *KobHLH* members were distributed across subfamilies 1, 3–12, 14–19, and 22, with counts of 2, 7, 1, 2, 5, 5, 7, 9, 7, 4, 12, 8, 14, 6, 5, 17, 6, and 1, respectively. All assignments were supported by ultrafast bootstrap values ≥ 87% (1000 replicates, Figure 3). Five subfamilies (2, 13, 20, 21, and 23) contained only *AtbHLH* members. Notably, the total number of *bHLH* genes was comparable between the two species, implying that *K. obovata* and *A. thaliana* share a common ancestral *bHLH* gene set, and that lineage-specific expansion and losses occurred after their divergence.

### 3.4. Gene Structure and Exon–Intron Organization Analysis

**Figure 4:**
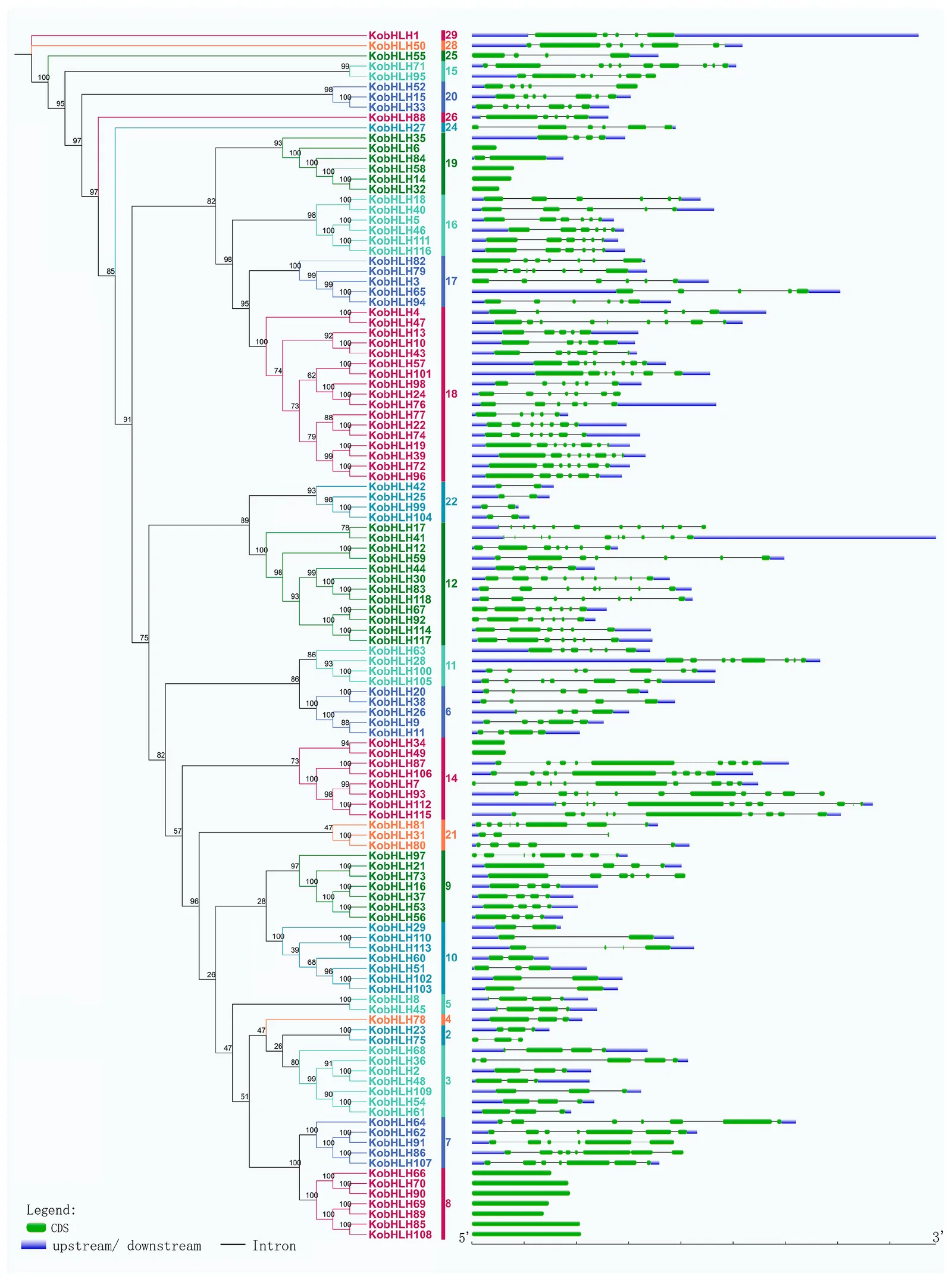
Phylogenetic relationships and gene structures of the *KobHLH* genes. Phylogenetic tree of *K. obovata* bHLH proteins (left panel). Exon–intron organization: yellow boxes (exons), blue boxes (UTRs) (right panel). Black lines (introns). Node labels indicate ultrafast bootstrap support values (1000 replicates).

To examine the structural features of the KobHLH family, we analyzed the exon–intron organization of the 118 *KobHLH* genes using their coding sequences. Overall, the *KobHLH* family genes were classified into 18 subfamilies, consistent with the phylogenetic classification described (Figure 4). Intron numbers varied from 0 to 10, and most members in the same subfamily exhibited comparable exon–intron structures (Figure 4). Interestingly, subfamilies S8 (seven members), S14 (two members), and S19 (four members) were entirely intron-less, while *KobHLH93* and *KobHLH17* had the highest intron count (10). Notably, over half of the *KobHLH* genes (66 members, 55.93%) contained 3-6 introns, with the most frequent counts being five (18 members) or six (22 members).

### 3.5. Conserved Domain and Motif Analysis of KobHLH Proteins

**Figure 5:**
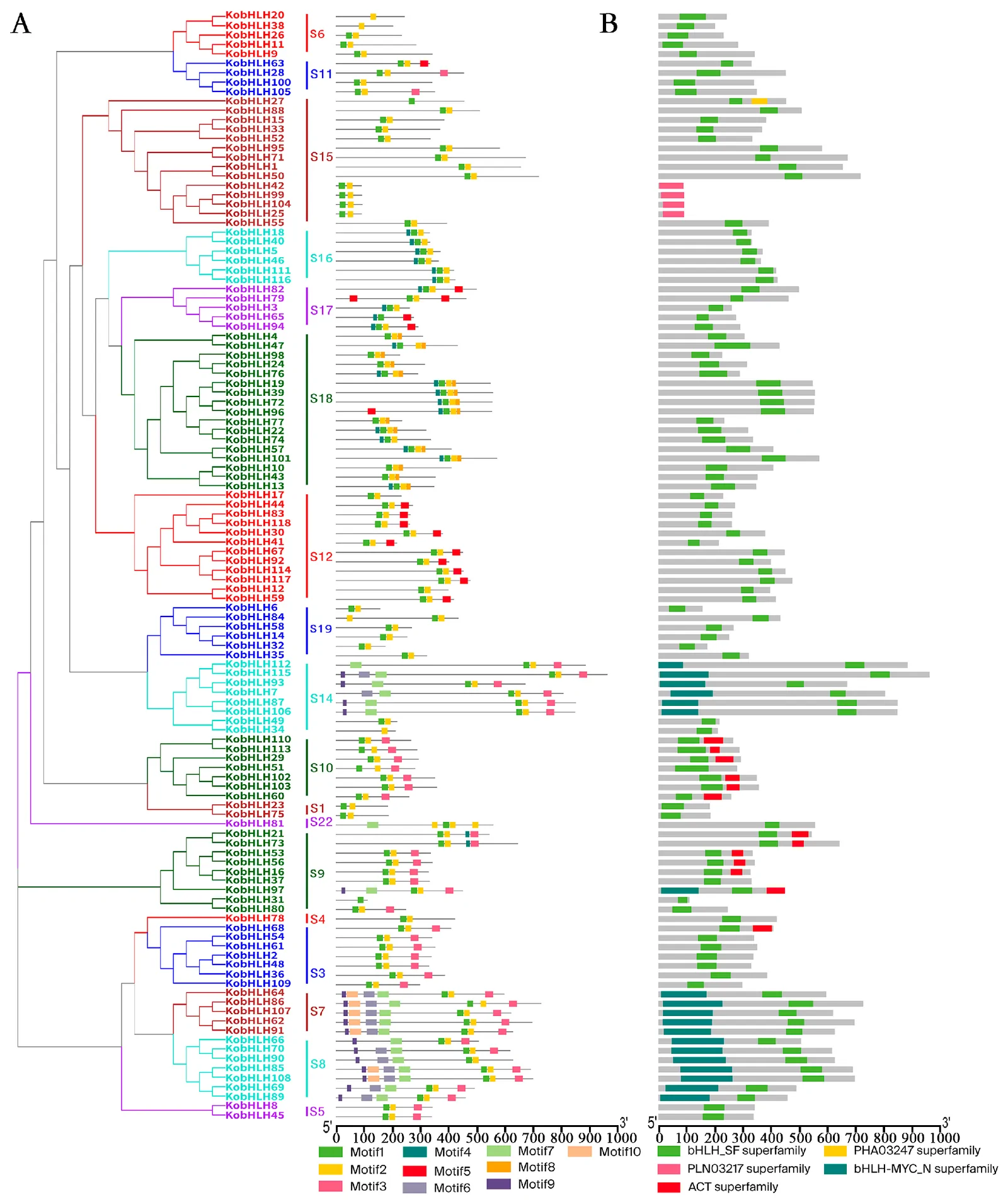
Gene motifs and conserved domains of the KobHLH proteins. (**A**): Ten conserved motifs (numbered 1–10) identified by MEME are shown in different colored boxes gray lines represent non-conserved sequences. (**B**): Five categories of conserved domains are shown in different colored boxes; domain positions along each protein are displayed from the N-terminus (**A**) to the C-terminus (**B**).

MEME analysis identified 10 conserved motifs in the 118 KobHLH proteins (Figure 5A). Members within the same subfamily shared similar motif compositions. KobHLH27 and KobHLH31 each contained only motif1 (M1), while KobHLH38 contained only motif2 (M2). KobHLH64 harbored the largest number of motifs, with the arrangement M9-M10-M6-M7-M1-M2-M3-M1. In addition, M1-M2 and M1-M2-M3 patterns were observed in 23.73% and 19.49% of the KobHLH genes, respectively. The N-terminal bHLH-MYC_N structure tended to contain at least one of M6, M7, and M9 (Figure 5B). With the exception of *KobHLH25*, *KobHLH42*, *KobHLH99*, and *KobHLH104*, which harbored the PLN03217 domain, all other KobHLH members carried the bHLH_SF domain (Figure 5B). Members containing either domain generally possessed at least M1 and M2 (Figure 5B). Furthermore, the PHA03247 and ACT domains were primarily localized to the C-terminal region of KobHLHs that contained M3 (Figure 5B).

**Figure 6:**
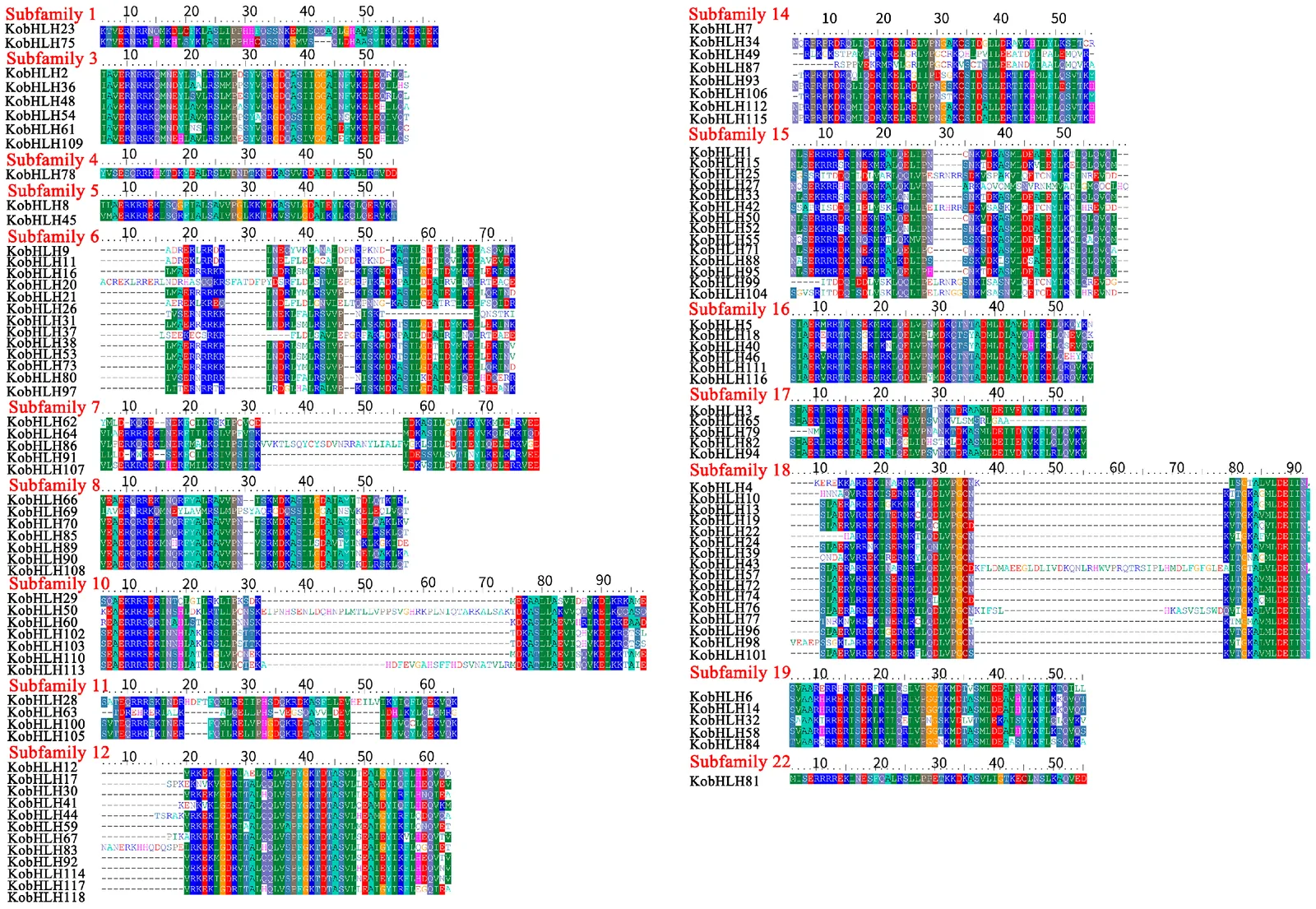
Multiple sequence alignment of the HLH domains of KobHLH proteins. Color shades represent residue conservation levels.

The C-terminal HLH region, approximately 40 amino acids in length, consisted of two alpha helices enriched in hydrophobic residues and is involved in regulating target gene expression across various signaling pathways [96,97]. To examine the phylogenetic relationships among KobHLH proteins, multiple sequence alignment was performed on their HLH domains, which spanned 118 amino acids (Figure 6). Notably, alignment based on the HLH domains alone provided clearer separation of the phylogenetic groups and subgroups.

### 3.6. Promoter Cis-Acting Element Analysis

**Figure 7:**
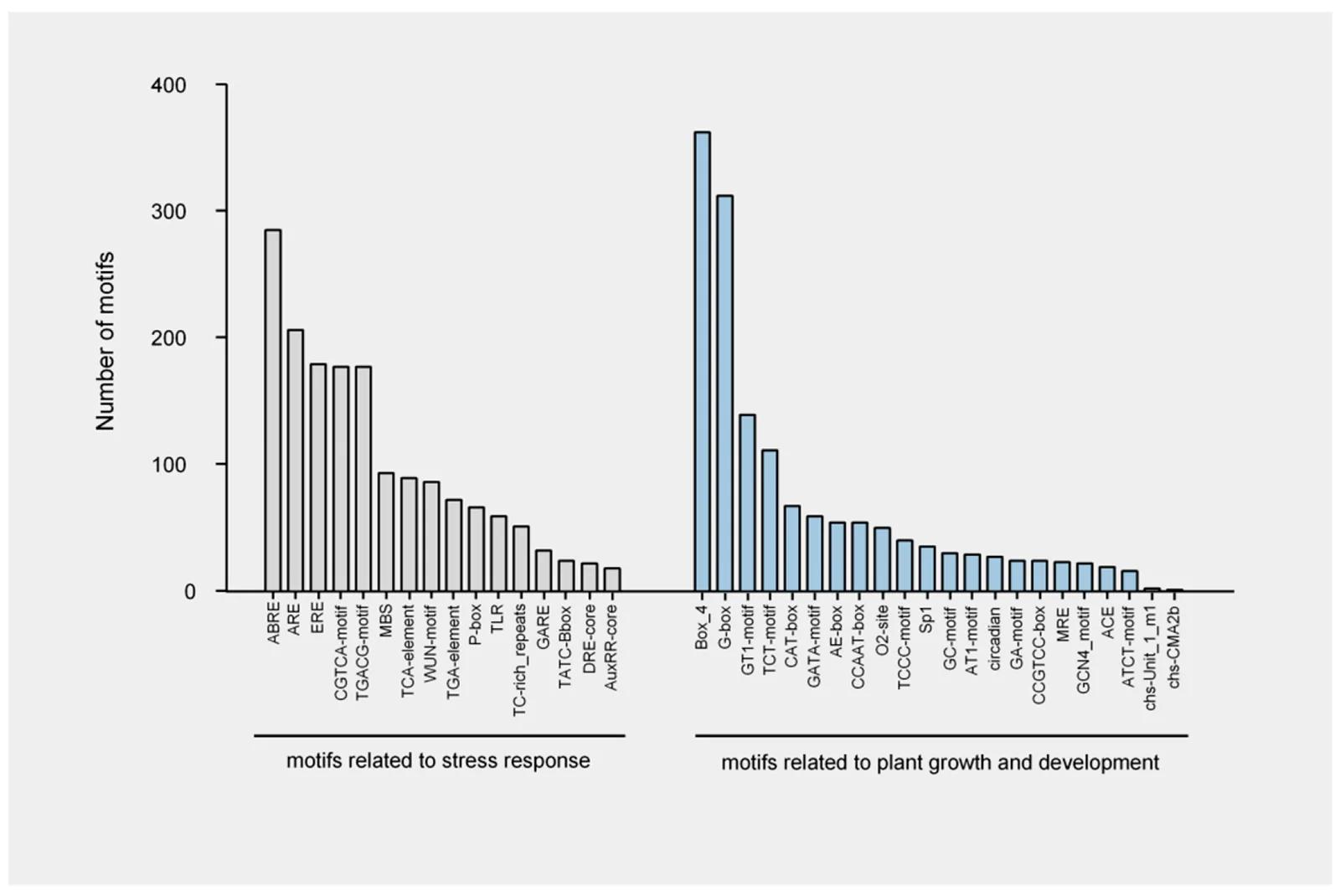
*Cis*-acting elements identified in the 2 Kb upstream promoter regions of *KobHLH* genes. Gray bars represent motifs related to stress response. Light blue bars represent motifs linked to growth and development.

*Cis*-acting elements in gene promoters are essential for the temporal, spatial, and cell type-specific regulation of gene expression [93]. Here, we used PlantCARE to analyze the 2 Kb upstream promoter regions of each *KobHLH* gene for cis-acting regulatory elements. The identified motifs were classified into stress response and development-related categories. The stress-related group comprised phytohormone response elements (ABRE, CGTCA-motif, ERE, TGACG-motif), environmental response elements (ARE, MBS, LTR, DRE core), and defense response elements (WUN-motif, TC-rich repeats) (Figure 7, Table S2). Among development-associated elements, light-responsive motifs (Box 4, G-box, GT1-motif, and TCT-motif) were predominant. The most abundant response elements were Box 4 (362, in 95 genes), G-box (312, in 95 genes), ABRE (285, 105 genes), and ARE (206, 99 genes). Notably, the *KobHLH95* promoter region contained the highest numbers of AREB (14) and G-box (18) sites, whereas the *KobHLH18* and *KobHLH80* promoters harbored the most ARE (6) and Box 4 (13) sites, respectively. The G-box element is the canonical binding target of bHLH transcription factors [12]. Thus, a total of 312 G-box motifs were identified in the promoters of 95 *KobHLH* genes, suggesting that these *KobHLH* genes may be subject to bHLH TFs-mediated regulation, potentially through interactions involving other KobHLH members. Nevertheless, experimental approaches such as yeast one-hybrid (Y1H) or electrophoretic mobility shift assays (EMSA) are required to confirm direct binding and regulatory activity.

### 3.7. Collinearity and Gene Duplication Analysis

To investigate syntenic relationships, we further conducted a collinearity analysis of *bHLH* genes among *K. obovata*, *A. thaliana*, and *P. trichocarpa* (Figure S1). A total of 159 orthologous gene pairs were identified between *K. obovata* and *A. thaliana*, and 207 pairs between *K. obovata* and *P. trichocarpa*, with genes distributed across all chromosomes. Synteny analysis revealed that *KobHLH25* and *KobHLH107* each formed four homologous pairs in *A. thaliana* (Figure S1A) and three homologous pairs in *P. trichocarpa* (Figure S1B). In addition, *KobHLH10*, *KobHLH13*, *KobHLH43*, and *KobHLH86* each formed three homologous pairs in both species (Figure S1). *KobHLH10*, *KobHLH13*, *KobHLH25*, *KobHLH43*, *KobHLH86*, and *KobHLH107* exhibited high collinearity conservation across *K. obovata*, *A. thaliana*, and *P. trichocarpa*, suggesting their retention following whole-genome duplication events.

To assess the contribution of gene duplication to *KobHLH* family expansion, tandem and segmental duplication events were analyzed. No tandem duplication events were detected among the 118 KobHLH genes. In contrast, 74 segmental duplication pairs involving 90 KobHLH members (~76% of the family) were identified (Figure S2). The segmental duplication relationships were distributed across multiple chromosomes, with chromosomes 2 and 8 serving as major duplication hubs. These results indicate that segmental duplication, rather than tandem duplication, has been the primary driver of *KobHLH* gene family expansion.

### 3.8. Expression Profiling of KobHLH Genes Across Tissues

**Figure 8:**
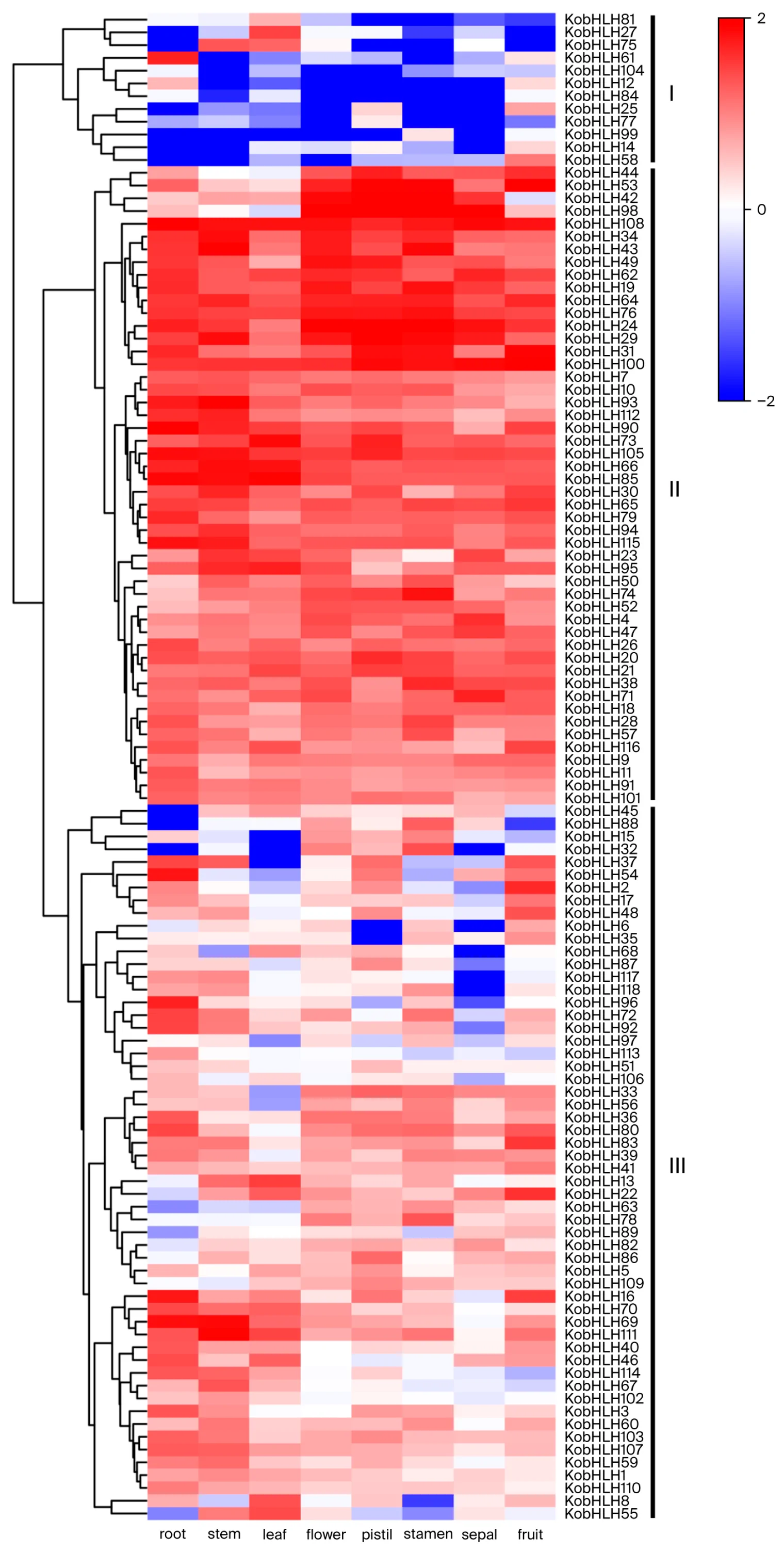
Expression patterns of *KobHLH* genes in different tissues. FPKM values were log10-transformed and z-score normalized. Red or blue represent high and low relative expression levels, respectively, with color intensity indicating the degree of up- or downregulation.

To explore the possible functional specificity, we examined their transcript abundance in eight different tissues (root, stem, leaf, flower, pistil, stamen, sepal, and fruit) using RNA-seq data from NCBI (accession PRJNA678025). Based on hierarchical clustering of expression profiles, the 118 *KobHLH* genes were classified into three groups (Figure 8 I to III). Group I, comprising 12 members, was characterized by consistently low expression across all tissues (Figure 8 I). Group II, comprising 50 members, exhibited strongly correlated expression patterns among tissues, suggesting coordinated regulation (Figure 8 II). Group III, comprising 56 members, showed diverse expression patterns in various tissues (Figure 8 III). For example, *KobHLH45* and *KobHLH88* were minimally expressed in roots, while *KobHLH54*, *KobHLH96*, *KobHLH16*, and *KobHLH69* were highly expressed (Figure 8 III). In addition, *KobHLH111* was preferentially expressed in stems, whereas *KobHLH15*, *KobHLH32*, and *KobHLH37* had low leaf expression (Figure 8 III). These results suggest considerable functional diversification within the *KobHLH* family.

### 3.9. Salt-Responsive Expression Profiles of KobHLH Genes

**Figure 9:**
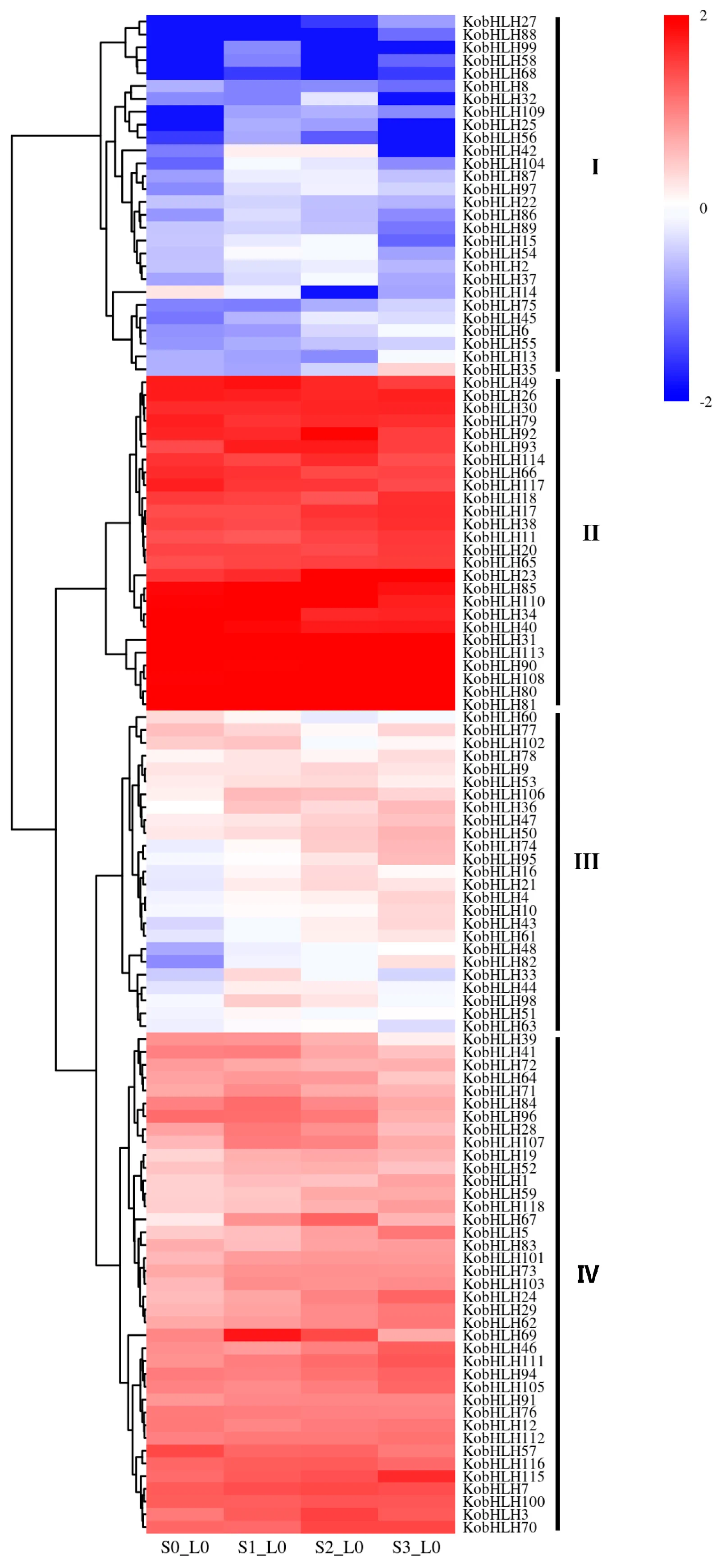
Salt−responsive expression profiles of *KobHLH* genes. PFAM values from RNA−seq data were log10−transformed and z−score normalized. S0_L0, S1_L0, S2_L0, and S3_L0 represent 0‰, 10‰, 20‰, and 30‰ salinity treatments, respectively. Red or blue represent high and low relative expression, respectively.

To explore the transcriptional dynamics of *KobHLH* genes under salt stress, gene expression was investigated based on transcriptome data (PRJNA1051781). Hierarchical clustering classified the 118 *KobHLH* genes into four groups based on their responsiveness to salinity (Figure 9 I–IV). Group I, comprising 28 *KobHLH* members, exhibited consistently low expression across all salinity levels (Figure 9 I). Group II, comprising 26 *KobHLH* members, exhibited strong constitutive expression (Figure 9 II). Group III, comprising 25 *KobHLH* members, displayed significant up- or downregulation relative to the control under salt stress (Figure 9 III). Finally, group IV contained 39 *KobHLH* members with relatively high gene expression levels (Figure 9 IV).

**Figure 10:**
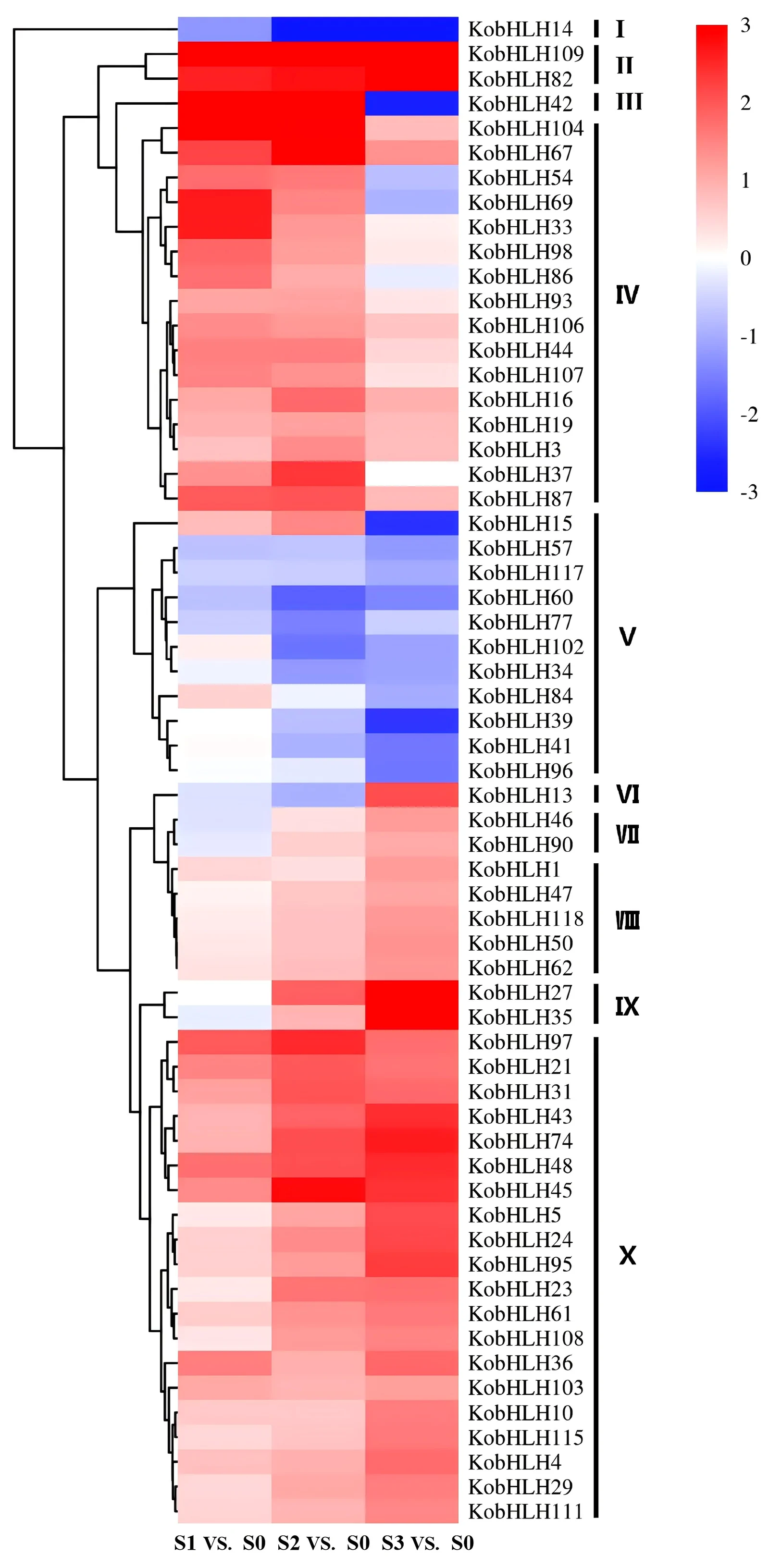
Expression pattern of differentially expressed *KobHLH* genes under salt stress. Heatmap based on log_2_ (fold change) values (z−score normalized). S0, S1, S2, and S3 denote 0‰, 10‰, 20‰, and 30‰ NaCl treatments, respectively. Red or blue represent upregulation and downregulation, respectively.

To further examine genes strongly associated with salt stress, we identified DEGs from the combined set of pairwise comparisons (S1 vs. S0, S2 vs. S0, and S3 vs. S0) for further analysis. The DEGs were subsequently resolved into 10 clusters (Figure 10 I to X) based on temporal expression patterns across salt gradients (Figure 10 I–X). Specifically, *KobHLH14* (cluster I) was downregulated across all salinity levels (Figure 10 I). Members of group II (*KobHLH109* and *KobHLH82*) were upregulated at all salinity levels (Figure 10 II). In group III, *KobHLH42* was upregulated under low and medium salinity but downregulated under high salinity (Figure 10 III). Group IV, comprising 16 *KobHLH* genes, showed general upregulation except for *KobHLH54* and *KobHLH69*, which were downregulated under high salt (Figure 10 IV). Group V, comprising 11 *KobHLH* genes, was inhibited under high salt, with repression reaching up to 2.4-fold (Figure 10 V). In group VI, *KobHLH13* was repressed by low and medium salt but activated by high salt. (Figure 10 VI). In group VII, *KobHLH46* and *KobHLH90* were repressed under low salt but activated by medium and high salt (Figure 10 VII). Group VIII displayed a positive correlation between expression and increasing salt concentration (Figure 10 VIII). Cluster IX (*KobHLH27* and *KobHLH35*) mirrored the pattern of cluster VII, also showing a positive correlation with salinity (Figure 10 IX). Group X, comprising 20 *KobHLH* genes, was consistently upregulated in response to salt (Figure 10 X). Overall, the pronounced differential expression of these genes under salinity stress highlights their potential as key regulators in the salt-stress response of *K. obovata*.

### 3.10. Co-Expression Network of Salt-Responsive KobHLH Genes

To explore potential functional associations among salt-responsive *KobHLH* members and other DEGs, we constructed a co-expression network using the 118 *KobHLH* genes as bait. The final co-expression network contained 20 nodes and 198 edges, predominantly enriched in metabolic pathways and biosynthesis of secondary metabolites (Figure S3), corroborating the KEGG enrichment results (Figure S4). *KobHLH108* and *KobHLH5* were the most highly connected nodes, followed by *KobHLH24* and *KobHLH115*. However, *KobHLH3*, *KobHLH29*, and *KobHLH39* exhibited only one or two connections each. These connectivity patterns suggest that *KobHLH108*, *KobHLH5*, *KobHLH24*, and *KobHLH115* serve as hub genes in the salt-stress response, warranting further experimental validation.

It should be noted that a highly stringent threshold (|r| > 0.98) was applied to minimize false positives. Consequently, the network represents only the most robust co-expression relationships and may not capture the full spectrum of salt-induced regulatory interactions involving *KobHLH* genes.

## 4. Discussion

As one of the largest transcription factor families in plants, bHLH TFs play key roles in a large number of biological processes related to growth, development, and stress response [98]. Since their initial isolation in tomato, the study of bHLH TFs has expanded considerably with the availability of sequenced genomes from *A. thaliana*, poplar, rice, moss, and algae [17,99]. *K. obovata*, a highly stress-resistant mangrove species widely used in coastal wetland ecological restoration [100], has remained unexplored in this regard. In this study, we identified 118 *bHLH* genes across the *K. obovata* genome and observed a progressive increase in the number of bHLH genes from chlorophyte algae to dicotyledonous plants (Figure 2), which is consistent with increasing organismal complexity [101]. In *K. obovata*, tandem and segmental duplication analyses revealed that segmental duplication events have contributed more substantially to *KobHLH* family expansion than tandem duplication, suggesting that ancient whole-genome duplication events were the primary source of KobHLH family expansion. According to phylogenetic analysis, the majority of *bHLH* subfamilies contained members from *A. thaliana and K. obovata*, whereas a few subfamilies were species-specific. This suggests a common ancestral origin followed by lineage-specific differentiation after separation.

Genomic distribution was uneven, with most *KobHLH* genes concentrated on chromosomes 1, 2, 3, 4, and 8 (Figure 1), implying potential functional clustering. Subcellular localization prediction indicated that the majority of KobHLH proteins are localized to the nucleus (Table S1), consistent with their role as transcription factors. In addition, the 118 *bHLH* genes differed considerably in length, reflective of genomic complexity (Table S1), indicating functional heterogeneity (Table S1). Moreover, the KobHLH proteins contained diverse conserved motifs, with members of the same subfamily tending to share similar motif compositions (Figure 5), reflecting both conservation and diversification. According to a prior study, a non-bHLH domain may serve various molecular roles [101]. Consistently, many KobHLH proteins harbored additional non-bHLH domains (Figure 5), suggesting functional heterogeneity among family members. Currently, the biological roles of *bHLH* genes in *K. obovata* remain largely unexplored, and the functions of specific amino acids in KobHLH proteins are poorly characterized. Further studies are therefore warranted to explore the functional diversity of KobHLH genes and proteins.

*Cis*-acting elements in gene promoters are essential for the initiation of gene transcription, and their composition can shape expression patterns [102]. In our study, a total of 38 cis-elements were identified in the *KobHLH* promoter regions (Figure 7, Table S2). These included elements linked to phytohormones, cold, drought, and dehydration, and defense responses (Table S2), as well as motifs for light responsiveness, meristem-specific activation, circadian regulation, and other developmental processes (Table S2). This broad repertoire of *cis*-regulatory elements in the *KobHLH* promoter regions indicated that these genes are poised to respond to environmental and developmental cues and also reflect the transcriptional-level functional diversification.

The bHLH TFs are known to participate in both biotic and abiotic stress responses. AtbHLH116 (ICE1) in *A. thaliana* improves cold tolerance by activating COR genes [47]. *AtbHLH92* contributes to osmotic stress, and *AtbHLH17* (*AtAIB*) enhances drought resistance through ABA signaling [51,55]. In rice, *OsbHLH006* and *OsbHLH148* confer drought tolerance via the jasmonic acid (JA) pathway [58]. In tomato, *SlICE1a* can enhance resistance to cold, osmotic, and salt stress. TabHLH39 in wheat is involved in freezing, salt, and drought tolerance [59], and overexpression of *PtrbHLH186* in poplar increases drought tolerance [60]. We analyzed the expression patterns of *KobHLH* genes under salt stress (Figure 9 and Figure 10) and identified 61 DEGs, including *KobHLH27*, *KobHLH69*, and *KobHLH115* (Figure 10). These results suggest that KobHLH TFs may have undergone functional differentiation under salt stress, a possibility that warrants further targeted validation.

Genes sharing similar structures tend to be grouped into the same subfamilies and perform analogous biological roles. For example, in *A. thaliana*, *ANAC002/ATAF1* and *ANAC072/RD26* belong to the same subfamily, and both are involved in drought stress response [59,60]. *PtrNAC006*, *PtrNAC007*, and *PtrNAC120* in poplar are linked to drought resistance [64]. *AtbHLH17* (*AtAIB*), *AtbHLH92*, and *AtbHLH122* have all been reported to function in drought tolerance [51,55,103]. Furthermore, *AtbHLH106*, *AtbHLH112*, and *MYC2* (*AtbHLH6*) are known to be closely related to salt tolerance [104,105,106,107,108]. Therefore, orthologous clusters in the same branch share conserved functions. In our salt-stress expression analysis, the *KobHLH* genes (*KobHLH102*, *KobHLH103*, *KobHLH67*, *KobHLH117*, *KobHLH69*, and *KobHLH108*), which were clustered with *AtbHLH106*, *AtbHLH112*, and *MYC2* (*AtbHLH6*) in *A. thaliana,* were all upregulated under salinity. Moreover, *KobHLH67* and *KobHLH108* also served as hub nodes in the co-expression network, reinforcing their candidacy as salt-responsive regulators.

As this study is purely bioinformatic and transcriptomic, lacking wet-lab functional validation, we applied an integrated prioritization scheme to identify candidate genes for future functional studies. The scheme incorporated three criteria: (i) significant differential expression (|log_2_FC| ≥ 2, q < 0.05) under at least two salinity levels, (ii) hub status in the co-expression network, and (iii) phylogenetic clustering with functionally validated salt-responsive *bHLH* genes (*AtbHLH106*, *AtbHLH112*). Genes meeting all three criteria were assigned the highest priority. By this approach, *KobHLH108*, *KobHLH67*, and *KobHLH5* were identified as the top candidates, which warrant further experimental validation using methods such as qRT-PCR, heterologous overexpression, or yeast one-hybrid assays.

The molecular mechanisms by which bHLH TFs mediate salt adaptation are increasingly well understood. In *Arabidopsis*, *AtMYC2* and *AtbHLH122* directly regulate the vacuolar Na^+^/H^+^ antiporters *AtNHX1* and *AtNHX6*, thereby promoting Na^+^ compartmentalization [109]. Similarly, CabHLH035 from pepper binds to the *SOS1* promoter to enhance Na^+^ extrusion and activates *P5CS* to drive proline biosynthesis for osmotic adjustment [110]. Proline metabolism itself is fine-tuned by AtMYC2, which negatively regulates P5CS1 transcription under salt stress [108]. In parallel, bHLH TFs contribute to ROS detoxification through multiple pathways. *PagbHLH35* enhances enzyme genes (POD, SOD) and reduces H_2_O_2_ accumulation in transgenic poplar [111], while *BvbHLH93* from sugar beet directly upregulates SOD and POD and simultaneously suppresses ROS-producing *RbohD/F* genes [112]. *NtbHLH123* activates NADPH oxidase *NtRbohE* to generate a controlled ROS signal that triggers downstream responses [113]. Additionally, bHLH TFs are well-established regulators of secondary metabolism, particularly the flavonoid pathway. *AmDEL* from *Antirrhinum* coordinately upregulates flavonoid biosynthetic and ROS-scavenging genes, enhancing both flavonoid accumulation and salt tolerance in *Arabidopsis* [114].

Several lines of evidence suggest that the salt-responsive *KobHLH* candidates may operate through analogous regulatory circuits in *K. obovata*. First, the promoters of salt-responsive *KobHLH* genes are enriched in ABRE elements (Figure 7), implying regulation via the ABA signaling pathway, a central hub that coordinates ion transport, osmotic adjustment, and ROS scavenging under salinity [115,116]. Second, KEGG enrichment of the co-expression network highlighted biosynthesis of secondary metabolites, and hub genes including *KobHLH108* and *KobHLH5* were associated with these pathways, suggesting a potential role in regulating flavonoid-type stress metabolites, reminiscent of *AmDEL* [114] and *EbbHLH80* [117]. Third, the presence of ARE (anaerobic response element) and TC-rich repeat (defense response) in *KobHLH* promoters is consistent with a function in ROS detoxification, as these motifs are frequently linked to antioxidant gene regulation. Fourth, *KobHLH108* and *KobHLH67*, identified as co-expression hubs, are phylogenetically clustered with *AtbHLH106* and *AtbHLH112* (Figure 3), both of which are functionally linked to salt tolerance via ion homeostasis and ABA-dependent signaling [106,107]. Collectively, these observations suggest that the salt-responsive KobHLH TFs, particularly KobHLH108 and KobHLH67, may contribute to salt adaptation in *K. obovata* by coordinating ABA-mediated signaling, ion homeostasis, ROS scavenging, and secondary metabolism. Functional validation of these candidates via heterologous overexpression, knockout, or ChIP-seq will be required to confirm the proposed regulatory hierarchies.

Several limitations of the co-expression analysis should be acknowledged. First, the stringent correlation threshold (|r| > 0.98), although effective in reducing false positives, may have excluded biologically relevant but weaker co-expression relationships. Second, co-expression alone cannot establish regulatory causality; thus, the identified hub genes are candidates that require further experimental validation, such as yeast two-hybrid assays, bimolecular fluorescence complementation (BiFC), or ChIP-qPCR. Nevertheless, the consistency between the co-expression network and KEGG enrichment results provides orthogonal evidence supporting the involvement of the identified hub genes in salt-responsive pathways. Notably, *KobHLH108* and *KobHLH5* were among the nodes with the highest connectivity and were both linked to secondary metabolite biosynthesis, reinforcing the known function of bHLH TFs in stress-related metabolic reprogramming.

## Data Availability

All data that support the findings of this study are available from the corresponding author upon reasonable request.

## Supplementary Materials

The following supporting information can be downloaded at: https://www.mdpi.com/article/10.3390/life16081247/s1, Table S1: Details of the bHLH proteins in *K. obovata*; Table S2: Motifs of *K. obovata* bHLH proteins; Table S3: One-to-one orthologous relationships between *K. obovata* and A. *thaliana*; Table S4: The FPKM values of *bHLH* genes in different tissues; Table S5: The FPKM values of *bHLH* genes in response to salt treatment; Table S6: List of bHLH gene family protein accession numbers; Figure S1: Collinearity analysis of *bHLH* genes in different species; Figure S2: Segmental duplication relationships among *KobHLH* genes in *K. obovata*; Figure S3: Co-expression networks of salt-responsive *KobHLH* genes; Figure S4: KEGG pathway enrichment analysis of *KobHLH* genes in *K. obovata*.

## Abbreviations

The following abbreviations are used in this manuscript:

bHLH	Basic helix–loop–helix
TFs	Transcription factors
E-box	Enhancer-box
PIs	Isoelectric points
MWs	Molecular weights
ML	Maximum likelihood
MEME	Multiple Em for Motif Elicitation
