# Supplementary material for: Genome-Wide Identification and Characterization of bHLH Family in Kandelia obovata Under Salt Stress
Source: Life (Basel). 2026 Jul 28;16(8):1247. doi: 10.3390/life16081247 (PMC13514259; doi:10.3390/life16081247)
Supplement: Supplementary file 1 [file life-16-01247-s001.zip › Supplementary figures.pdf]

Supplementary Figure S1

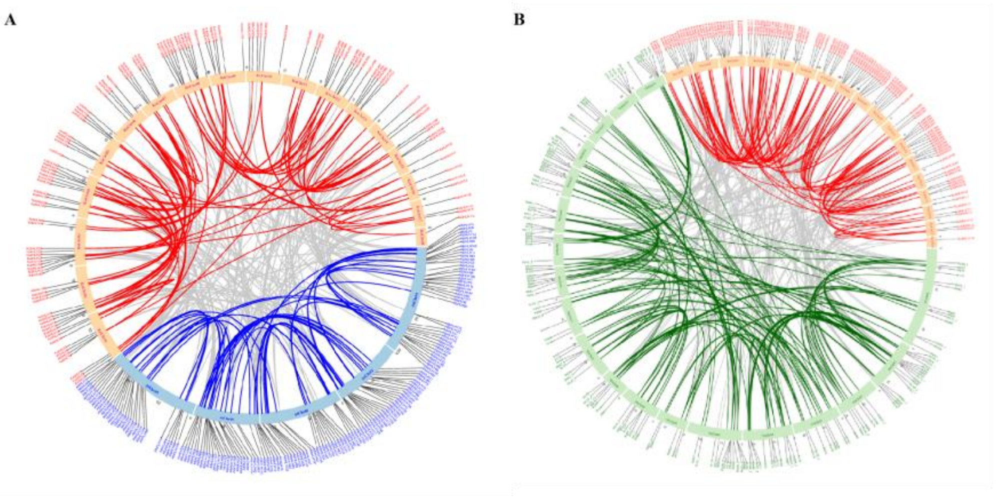

Supplementary Figure S1. Collinearity analysis of *bHLH* genes in different species. (A) Collinearity analysis of bHLH genes between *K. obovata* and *A. thaliana.* (B) Collinearity analysis of *bHLH* genes between *K. obovata* and *P. trichocarpa.* Red lines indicate *KobHLH* pairs in *K. obovata*. Blue lines indicate *AtbHLH* pairs in *A. thaliana*. Green lines indicate *PtrbHLH* pairs in *P. trichocarpa*. Gray lines indicate collinear blocks within *K. obovata* and other plants. Collinear pairs were identified by MCScanX based on gene order and sequence similarity. A higher density of connecting lines reflects greater genomic synteny rather than functional conservation.

Supplementary Figure S2

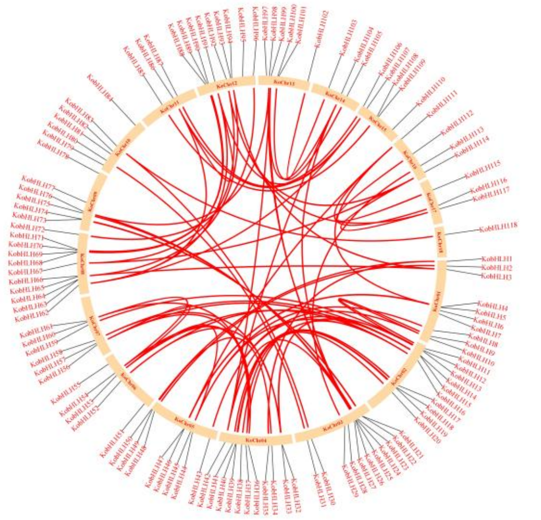

Supplementary Figure S2. Segmental duplication relationships among *KobHLH* genes in *K. obovata*. The outer ring displays the 18 chromosomes (KoChr01-KoChr18) in beige. KobHLH gene names are labeled radially. Red lines indicate segmental duplication gene pairs identified by MCScanX within collinear blocks. A total of 74 segmental duplication gene pairs involving 90 KobHLH members (~76% of the family) are shown.

Supplementary Figure S3

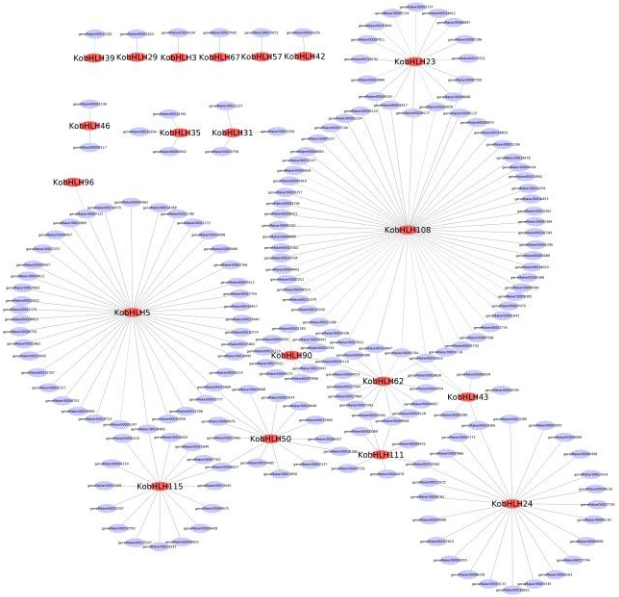

Figure S3. Co-expression networks of salt-responsive *KobHLH* genes. Each node represents a gene in a network. Co-expression pairs were filtered at $|r| > 0.98$ and $p < 0.001$. The network therefore depicts only the strongest transcriptional correlations among salt-responsive DEGs.

Supplementary Figure S4

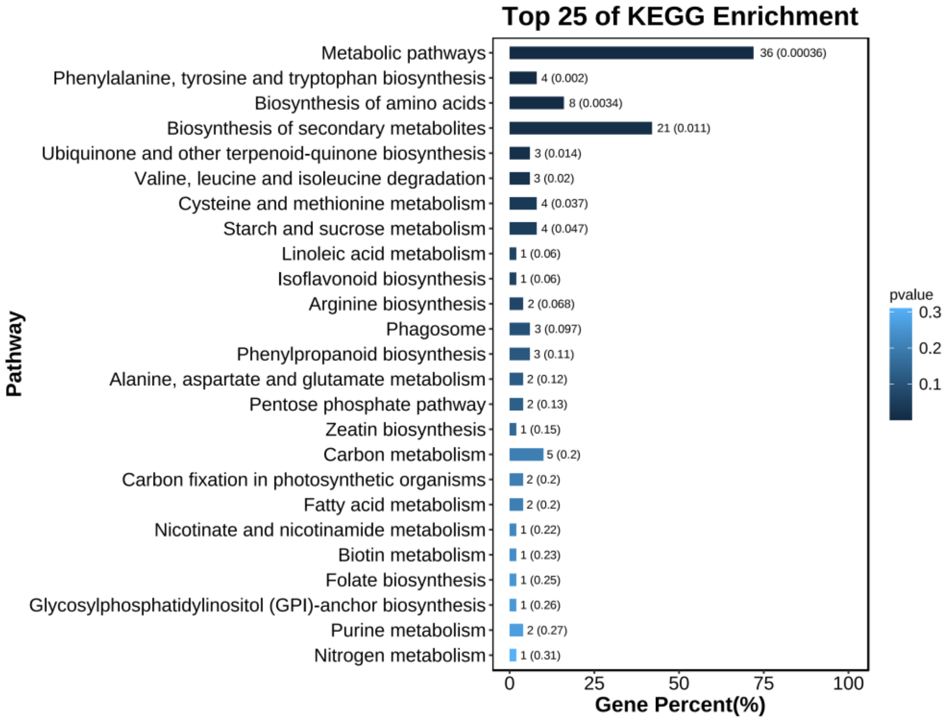

Supplementary Figure S4. KEGG pathway enrichment analysis of *KobHLH* genes in *K. obovata.* The horizontal bar chart shows the top 25 enriched pathways. The x-axis represents the percentage of genes mapped to each pathway. Numbers inside each bar indicate the gene count and the corresponding pvalue (hypergeometric test). Bar color denotes the pvalue according to the gradient scale on the right. The eight pathways with $p < 0.05$ were considered significantly enriched.
